# *Fosab*, but not *fosaa*, plays important role in learning and memory in fish—insights from zebrafish gene knockout study

**DOI:** 10.3389/fcell.2025.1503066

**Published:** 2025-01-27

**Authors:** Qiuling Wang, Lixin Zhang, Chenyuan Zhu, Ke Lu, Jiaqi Wu, Xu-Fang Liang

**Affiliations:** ^1^ College of Fisheries, Huazhong Agricultural University, Wuhan, Hubei, China; ^2^ Engineering Research Center of Green development for Conventional Aquatic Biological Industry in the Yangtze River Economic Belt, Ministry of Education, Wuhan, China; ^3^ Hubei Hongshan Laboratory, Fishery College, Huazhong Agricultural University, Wuhan, China

**Keywords:** fosaa, fosab, learning and memory, brain development, zebrafish

## Abstract

**Introduction:** Learning and memory allow individuals to adapt to their environmental needs and survive. Fish have the ability to solve complex learning tasks, associative learning, and flexible spatial memory. The proto-oncogene fos (*c-fos*) has been reported to be involved in brain development, learning and memory in mammals. However, whether the c-fos plays a vital role in learning and memory in fish is unclear.

**Methods:** Almost all fish have two paralogues of *c-fos* named *fosaa* and *fosab*. We used CRISPR/Cas9 technology to generate *fosaa* and *fosab* knockout zebrafish models.

**Results:** In this study, we discovered the brain weight marked reduction in *fosaa* ^−/−^ and *fosab* ^−/−^ zebrafish compared with the wild-type (WT) (AB strain) zebrafish. In the T-maze behavioral assay, the *fosab* ^−/−^ zebrafish took significantly more than the average time to complete the assigned trial as the increase in the days compared to WT zebrafish, while the *fosaa* ^−/−^ zebrafish did not show a notable variance. The average time to complete the trial in *fosab* ^−/−^ zebrafish was significantly higher than in WT zebrafish. The relative mRNA expression level of c-*jun* in *fosab* ^−/−^ zebrafish was significantly higher than that in WT zebrafish, while the *fosaa* ^−/−^ zebrafish has no discernible trend. Additionally, the phylogenetic and multiple amino acid alignment results indicated that fish *fosab* has a higher identity with mammals Fos.

**Discussion:** By integrating the above results, we found that *fosab*, but not fosaa, may possess a learning and memory function in fish. For the first time, we illustrated the role of *fosaa* and *fosab* in learning and memory via *c-fos* knockout in fish, which can provide new insights into environmental adaptation.

## 1 Introduction

Learning and memory are essential to organism survival and are conserved across various species, especially vertebrates (Tan et al., 2022). Mammals are able to continually learn and acquire skills to respond to the demands of a changing environment (Barron, 2021). Proto-oncogene fos (*c-fos*), an immediate early response gene, provides an essential function in the regulatory component of the nuclear response to mitogens and other extracellular stimuli and is a candidate gene for the role of an adaptive regulator (Curran and Morgan, 1987; Randall et al., 1992). Adaptive regulators are proteins that modulate the expression of genes involved in the long-term response, while learning is one of the most representative examples of long-term alteration (Curran and Morgan, 1987). The *c-fos* gene is widely used as a functional marker of brain activity in terms of neuroscience (Ariyasiri et al., 2021; Cai et al., 2022; Kubra et al., 2022; Morris et al., 2000). Emerging evidence suggested *c-fos* mutant could impair neuronal excitability, brain development and long-term learning and memory in mice (Fleischmann et al., 2003; Kim et al., 2003; Paylor et al., 1994; Velazquez et al., 2015; Zhang et al., 2002). Previous studies have demonstrated that the dorsolateral telencephalon of fish could be homologous to the mammalian hippocampus sharing functional similarities (Gómez et al., 2020; Naderi et al., 2016). Fish also have the ability to solve complex learning tasks. However, there is a paucity of information on whether *c-fos* plays a vital role in learning and memory in fish.

Zebrafish are a valuable, useful and promising vertebrate model organism to study a wide range of biological disciplines, including neuroscience on account of their short reproductive cycle, rapid external development, high genetic homology to humans, and various tools available to investigate (de Abreu et al., 2019; Soussi-Yanicostas, 2022). The National Center for Biotechnology Information (NCBI)’s research and literature showed that almost all fish have two paralogues of *c-fos*, named *fosaa* and *fosab*, individually. The *fosaa* and *fosab* genes are highly expressed in the brain of zebrafish (Kubra et al., 2022; Maili et al., 2023). Thus, we identified and performed an evolutionary analysis of the *c-fos* gene from different vertebrates and used zebrafish to explore if the function of learning and memory is regulated by the *c-fos* (*fosaa* and *fosab*) gene in fish as it is in mammals.

Behavioral assays are broadly applied to evaluate the associative learning and memory abilities of mice and zebrafish, with the T-maze test being one of the most commonly employed methods (Bicakci et al., 2021; Kim J. et al., 2019; Kim Y.-H. et al., 2019; Song et al., 2022; Yoo et al., 2021b). Zebrafish also showed a color preference that could be targeted when designing color-based learning and memory experiments involving fear, anxiety, or reward in the zebrafish (Avdesh et al., 2012). Based on this, we assessed the function of the *fosaa* and *fosab* genes in learning and memory by combining color and food rewards to evaluate associative learning abilities in zebrafish between different groups.

The cholinergic system plays an important role in regulating learning and memory in mammals (Haam and Yakel, 2017). Cholinergic transmission is involved in memory formation and consolidation, even over long periods (Oda, 2010; Phillis, 2005). Acetylcholinesterase (AChE) participates at the end of cholinergic transmission based on the breakdown of acetylcholine in choline and acetic acid, and acetylcholine transferase (AChT) is involved in the production of acetylcholine (Ichikawa et al., 1997). Inhibition of AChE activity and upregulation of AChT activity will enhance cholinergic neurotransmission, which can improve impaired memory in mice (He et al., 2015; Nogami-Hara et al., 2018). The hyperglycemia promoted memory deficit in adult zebrafish, which results from increased AChE activity (Katiucia et al., 2014). Fos-immunoreactive cells were identified as neurons, predominantly clustered in areas corresponding to the regions exhibiting high acetylcholinesterase (AChE) activity (Liu et al., 1991). Whether the *fosaa* and *fosab* affect learning and memory through the cholinergic system in zebrafish is unclear.

Brain-derived neurotrophic factor (BDNF), a neurotrophin, promotes neuronal survival, differentiation, and growth (Ismail et al., 2021). BDNF is one of the upstream signals for *c-fos* expression and positively related to long-term memory formation and memory in rats (Bekinschtein et al., 2014; Yoo et al., 2021a). C-JUN, a component of the AP-1 (Activator Protein 1) transcription factor, regulates cell proliferation, differentiation, survival, and responses to stress and injury (Ganguly et al., 2013). Investigating how the *bdnf* and *c-jun* expression is altered in the *fosaa* and *fosab* knockout zebrafish offers valuable insights into the molecular mechanisms underlying learning and memory.

In the present study, we identified the *c-fos* genes and predicted phylogenetic and evolutionary relationships in several vertebrates. To investigate whether *c-fos* has the function of learning and memory, we generated *fosaa* and *fosab* knockout zebrafish models using CRISPR/Cas9 technology. Behavioral experiments, morphology of brain observation, and cholinergic system detection were utilized to evaluate the function. In addition, the mRNA expression of brain derived neurotrophic factor (*bdnf*) and jun proto-oncogene (*c-jun*) was measured.

## 2 Materials and methods

### 2.1 Sequence alignment and phylogenetic analysis of *c-fos* genes

*c-fos* gene sequences from various vertebrate species were retrieved from the National Center for Biotechnology Information (NCBI) (http://blast.ncbi.nlm.nih.gov/). Species included were: human (*Homo sapiens*), mouse (*Mus musculus*), chimpanzee (*Pan troglodytes*), chicken (*Gallus gallus*), pig (*Sus scrofa*), common frog (*Rana temporaria*), tropical clawed frog (*Xenopus tropicalis*), zebra finch (*Taeniopygia guttata*), rabbit (*Oryctolagus cuniculus*), vaquita (*Phocoena sinus*), West African lungfish (*Protopterus annectens*), gray bichir (*Polypterus senegalus*), coelacanth (*Latimeria chalumnae*), zebrafish (*Danio rerio*), medaka (*Oryzias latipes*), mummichog (*Fundulus heteroclitus*), Klamath speckled dace (*Rhinichthys klamathensis goyatoka*), large yellow croaker (*Larimichthys crocea*), oriental weatherfish (*Misgurnus anguillicaudatus*), minute mudskipper (*Periophthalmus magnuspinnatus*), channel catfish (*Ictalurus punctatus*), chum salmon (*Oncorhynchus keta*), Wuchang bream (*Megalobrama amblycephala*), snailfish (*Pseudoliparis swirei*), yellowtail (*Seriola aureovittata*), smalleye Pacific opah (*Lampris incognitus*), ringed pipefish (*Dunckerocampus dactyliophorus*), North African catfish (*Clarias gariepinus*), mandarinfish (*Synchiropus splendidus*), striped catfish (*Pangasianodon hypophthalmus*), European seabass (*Dicentrarchus labrax*), Asian seabass (*Lates calcarifer*), Gulf pipefish (*Syngnathus scovelli*), silver crucian carp (*Carassius gibelio*), dwarf seahorse (*Hippocampus zosterae*), grass carp (*Ctenopharyngodon idella*), southern bluefin tuna (*Thunnus maccoyii*), and swordfish (*Xiphias gladius*). Accession numbers of sequences are listed in Supplementary Table S1. Amino acid sequences of Fos were aligned using Clustal X, and phylogenetic analysis was performed with MEGA X. A neighbor-joining tree was constructed using the JTT model and evaluated through 1,000 bootstrap replicates.

### 2.2 Zebrafish maintenance and treatment

Adult wild-type (AB strain) zebrafish were purchased from the National Zebrafish Resource Center, Institute of Hydrobiology, Chinese Academy of Sciences (http://www.zfish.cn/). Wild-type and knockout zebrafish were maintained in the Chinses perch Center of Huazhong University with a recirculating water system (Meifengbishui Environmental Technology, China, Wuhan), the water at 26°C–28°C, the pH at 6.5–7.5, the dissolved oxygen at least 6.0 mg/L, and conductivity was 400–600 μs/cm. The fish were also given a 14-hour light/10-hour dark cycle. The recirculating water system was equipped with a sediment filter, post-carbon filter, fluorescent UV light, and sterilizing filter. Artemia was used to feed both the Larvae following near depletion of the yolk sac and adult zebrafish twice daily. The adult zebrafish were kept in tanks measuring 10 cm × 26 cm × 15 cm at a density 6 fish/L. All experimental procedures of zebrafish were approved by the Institutional Animal Care and Use Ethics Committee of Huazhong Agricultural University (Approval Number: HZAUFI-2020-0032).

### 2.3 Construction of zebrafish *fosaa* and *fosab* mutants by CRISPR/Cas9 technology

The wild-type (WT) zebrafish of AB strain was used to produce mutant lines. CRISPR/Cas9 technology was employed to generate *fosaa* and *fosab* zebrafish mutants. Single-guide RNAs (sgRNAs) were designed using the CCTOP tool (https://cctop.cos.uni-heidelberg.de/), and the sequences of the sgRNAs and PCR primers are listed in Table 1. sgRNAs were cloned into the pMD-18T vector and synthesized using the TranscriptAid T7 High Yield Transcription Kit (Thermo Fisher Scientific, Waltham, MA, United States). The night before microinjection, the male and female of the zebrafish were paired to produce 3-5 parents with well development and stable spawning. Female and male fish were separated by a partition, the partition was removed before microinjection on the second day, and the embryos were collected for 15 min after the parents had finished laying eggs. A mixture of sgRNA (50 ng/μL) and Cas9 protein (New England Biolabs, Ipswich, MA, United States) was co-injected into wild-type embryos at the 1–2 cell stage. The F0 generation was crossed with wild-type fish to produce F1 offspring. F1 heterozygotes zebrafish of *fosaa* and *fosab* were named *fos* ^*+/−*^ and *fosab* ^*+/−*^. F1 heterozygotes were then crossed to generate F2 homozygous mutants, and all experiments were conducted using F3 homozygous zebrafish. The same WT zebrafish stock was bred in parallel to generate the +/+ animals. *fosaa* and *fosab* mutants of zebrafish were named *fosaa* ^*−/−*^ and *fosab* ^*−/−*^ zebrafish. All of the experiments were conducted on the basis of known zebrafish genotypes in the study.

**TABLE 1:** sgRNAs and primer sequences for the quantitative real-time PCR.

Gene name Primer	Sequence of primer (5′to 3′)	Tm (℃)
*β -actin*	F CACCCTATGACAAGAGGAAGC	59
	R TGTGCCAGACGCCCAAG	
*fosaa* sgRNA1	GACACGGAGGTGATGATGGTCGG	58
*fosaa* sgRNA2	GCCTTGAGACGGAGACACGGAGG	58
*fosaa*	F CAGCGGAATTTATTATGTCCATCG	58
	R GCAGTGAATGCAGTATCTACCT	
*fosab* sgRNA1	GAAGAGATCGCCGTGACAGTTGG	58
*fosab* sgRNA2	GATTGAGCTGCGCCGTTGGAGGG	58
*fosab*	F CAGCAGACGAGCAAGGAAATA	58
	R CTTGCAGTGTATCGGTGAGTT	
*c-jun*	F GGACTTCTCAAACTGGCATCAC	58
	R AGGTGACGTTGGGCATGTG	
*bdnf*	F CGCCGTTACTCTTTCTCTTGGA	58
	R CCATTAGTCACGGGGACCTTC	

### 2.4 Growth performance and brain weight assay

Wild-type (WT), *fosaa* ^*−/−*^, and *fosab* ^*−/−*^ zebrafish were maintained at identical stocking densities and culture conditions. Length and weight were measured at 273 dpf (day post-fertilization) for each group. Six zebrafish of each genotype and theirs corresponding WT zebrafish were anesthetized with 80 mg/L MS-222 prior to measurement, placed on clean gauze to absorb surface water to measure length and weight. Then, the same fish were executed by decapitation, and the brains were carefully dissected, weighed, and preserved in formaldehyde fixative.

### 2.5 T-maze test

The T-mazed assay was conducted based on the protocol by (Colwill et al., 2005; Pilehvar et al., 2020), with modifications in apparatus size. The test tank consisted of a T-shaped transparent maze equipped with two removable color sleeves (green and red). A start box, measuring 10 cm × 10 cm × 10 cm, was located at the base of the maze and could be sealed off. Fish were fed only during the daily training sessions. The test spanned 25 days and comprised three phases: (ⅰ) Pre-training (days 1–2): a period for zebrafish to adapt with conditioning of the T-maze without the colour stimulus. They were given 2 min in the start box to acclimate. Once the fish exited, the start box door was closed, and Artemia was manually added as a reward upon entering the open arm. The delay interval prior to reward delivery following correct performance was approximately 3–5 s. The T-maze was rinsed and refilled after each trial to remove residual odors. The total time to and complete the trial were recorded using timer manually for each trial. (ⅱ) Discrimination (days 1–16): a period of 16 days for zebrafish taking choice between the two coloured arms (red and green) of T-maze. Each consisting of four trials with 30-second intervals. The position of colored sleeves alternated (RGGRGRRG). Correct choices were rewarded with food, while incorrect choices triggered a correction procedure where the incorrect arm was blocked, forcing the fish to choose the correct arm. Half of the fish (n = 6) were rewarded for choosing red, the other half for green. Time to leave the start box and enter an arm, as well as the colour of the arm chosen was recorded using timer manually in discrimination trials. (ⅲ) Extinction (days 1–7): a period of 7 days for zebrafish performing the same procedure as the discrimination training with two exceptions: no food reward and no correction rounds were given. The T-maze test was carried out at 280 dpf of each zebrafish group, and the behavioral experiments performed in same room as they were housed at 9:00 a.m. each day. The experimenters maintained a distance of 0.5 m from the fish during the experiment. A total of 48 zebrafish, including *fosaa* ^*−/−*^ zebrafish (n = 12), *fosab* ^*−/−*^ zebrafish (n = 12), and their corresponding WT zebrafish (n = 12), were tested, with food provided only during training sessions. The recorded time in three training phases was analyzed by SPSS 19.0 software Levene’s test or Kruskal-Wallis test.

### 2.6 Estimation of acetylcholinesterase (AChE) acetylcholine and acetylcholine transferase (AChT) activity in the brain of zebrafish

Three zebrafish of each genotype, and along with their corresponding WT zebrafish, were anesthetized with 80 mg/L MS-222 prior to decapitation. Subsequently, the brains were excised and placed in 1.5 mL tubes. The tissues were weighed, and saline was added at a ratio of 9:1 (volume: weight). Brains were mechanically homogenized in an ice-water bath at 2,500 r/min, followed by centrifugation for 10 min. The supernatant was collected to measure AChE and AChT activity using specific reagent kits (AChE, catalog no. A024-1-1; AChT, catalog no. A079-1) from Jian-cheng Institute of Biotechnology (Nanjing, China), following the manufacturer’s instructions. AChE activity was measured by quantifying the yellow product, TNB (Symmetric Trinitrobenzene), formed when AChE hydrolyzes acetylcholine. The absorbance was measured spectrophotometrically at 420 nm. AChT activity was determined using acetyl coenzyme A and choline as substrates, and the absorbance of the reaction products was measured at 324 nm.

### 2.7 RNA isolation and RT-PCR

Six zebrafish of each genotype, along with their corresponding WT zebrafish, were anesthetized with 80 mg/L MS-222 prior to decapitation. The brains were excised and placed in 1.5 mL tubes, and then stored at −80°C refrigerator for subsequent processing. Total RNA was isolated from zebrafish brain using the Trizol Reagent (TaKaRa, Tokyo, Japan) as per the manufacturer’s instructions and stored at −80°C. RNA purity was assessed using a BioTek Synergy™2 Multi-detection Microplate Reader (BioTek Instruments, Winooski, VT, United States) and agarose gel electrophoresis. cDNA synthesis was carried out using the HiScript II Q RT SuperMix reverse transcriptase (Vazyme, Piscataway, NJ, United States). Gene-specific primers for mandarin fish were designed with Primer 5.0 software and synthesized by Sangon (Shanghai, China). The sequences are listed in Table 1. *β-actin* was used as the housekeeping gene due to its stability (Zhang et al., 2016). Real-time qPCR was performed using a quantitative thermal cycler (BIO-RAD, United States) with a 20 μL reaction volume consisting of 10 μL AceQ qPCR SYBR Green Master Mix (Vazyme, United States), 8.2 μL double-distilled water, 1 μL template (cDNA), and 0.4 μL each of forward and reverse primers. PCR conditions were 95°C for 5 min, followed by 40 cycles at 95°C for 10 s, annealing at Tm for 30 s, and a melt curve step with acquisition every 6 s. Gene expression was calculated relative to *β-actin* using the comparative Ct (2^−ΔΔCt^) method. Results were expressed as mean ± SEM (n = 6).

### 2.8 Data analysis

Statistical analyses were performed using SPSS 19.0 software. Data were expressed as mean ± SEM. Homogeneity of variance was assessed using Levene’s test. When normality and equal variance between sample groups was achieved, the data were assessed using one-way ANOVA, and differences between group means were compared by Duncan’s post-hoc multiple comparison. When normality failed, Kruskal-Wallis test was performed, and followed by Dunn’s multiple comparisons test. Statistical significance was set at *P* < 0.05.

## 3 Results

### 3.1 Synteny analysis, sequence alignment and phylogenetic analysis of *c-fos* gens

**FIGURE 1:**
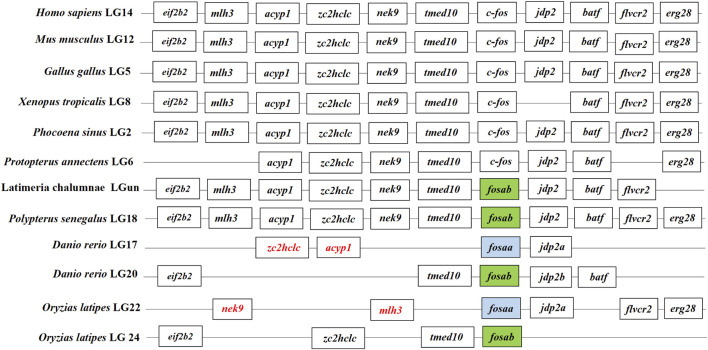
Synteny analysis of *c-fos* genes. The synteny analysis was performed by searching genes flanking *c-fos* in genomes of human (*Homo sapiens*), mouse (*Mus musculus*), chicken (*Gallus gallus*), tropical clawed frog (*Xenopus* tropicalis), Vaquita (*Phocoena sinus*), West African lungfish (*Protopterus annectens*), Gray bichir (*Polypterus senegalus*), coelacanth (*Latimeria chalumnae*), zebrafish (*Danio rerio*), medaka (*Oryzias latipes*). The color of the font in the box was marked in red indicated that the change in gene location.

**FIGURE 2:**
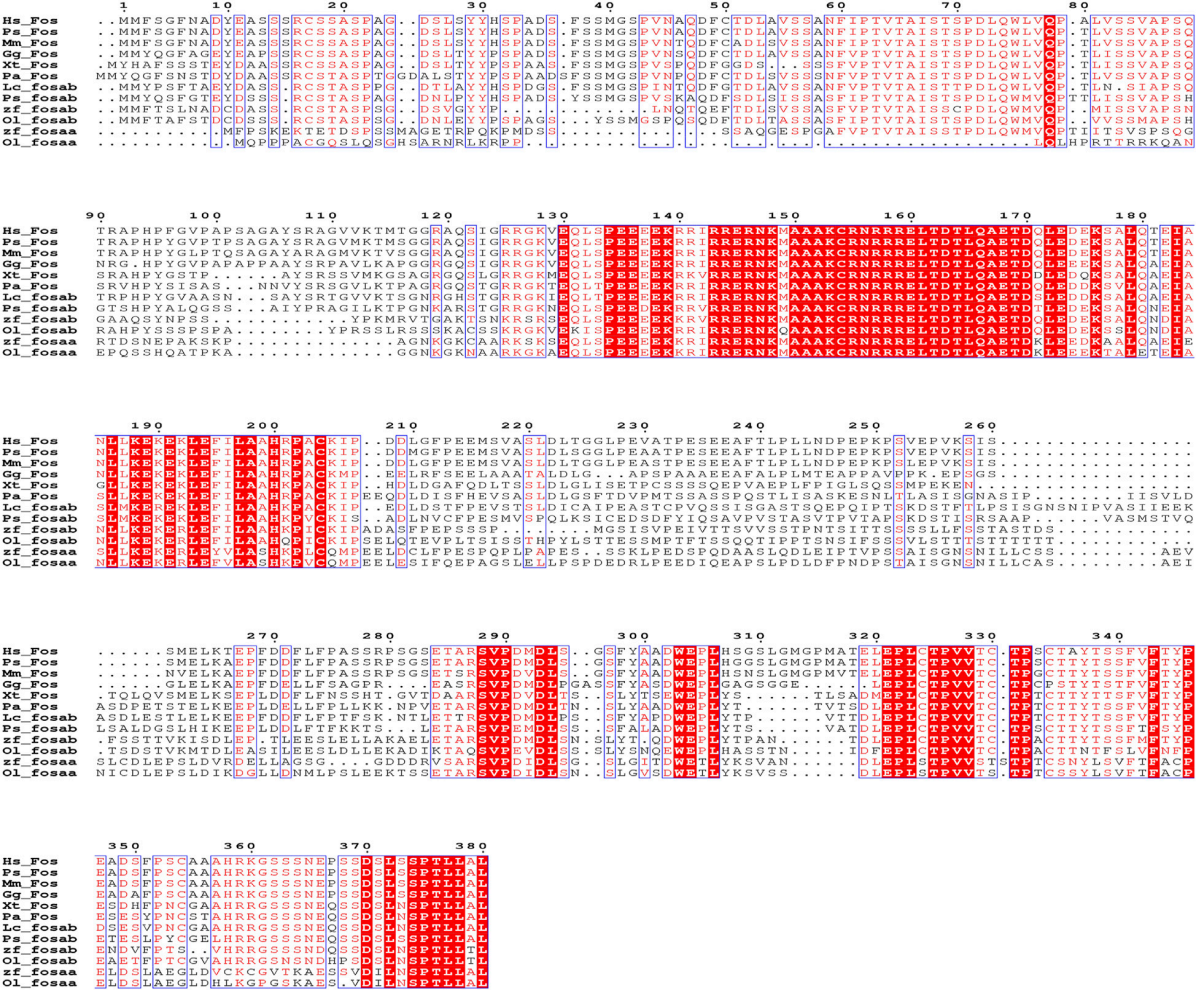
Sequence alignment of human (*Homo sapiens*, Hs), Vaquita (*Phocoena sinus*, Ps), mouse (*Mus musculus*, Mm), chicken (*Gallus gallus*, Gg), tropical clawed frog (*Xenopus tropicalis*, Xt), West African lungfish (*Protopterus annectens*, Pa), coelacanth (*Latimeria chalumnae*, Lc), Gray bichir (*Polypterus senegalus*, Ps), zebrafish (*Danio rerio*, Dr) and medaka (*Oryzias latipes*, Ol). Red with white letters represents fully conserved residues.

**FIGURE 3:**
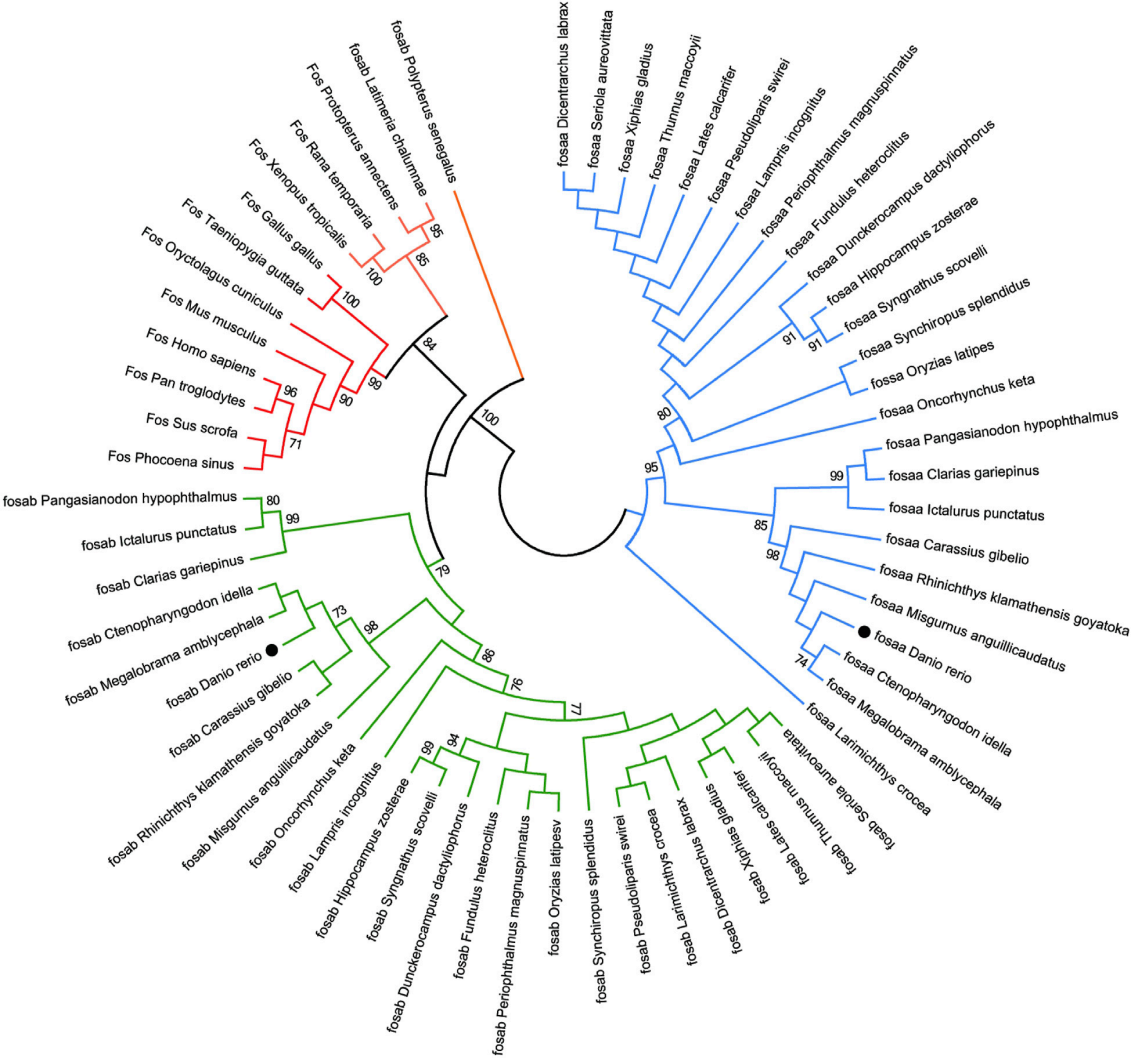
The Neighbor-joining tree based on the Fos proteins. The tree was built by the JTT model bootstrap method with 1,000 replications. The lines with the same color (red, blue or green) belong to one branch. The zebrafish *fosaa* and *fosab* were marked with a black dot.

We found that *c-fos* has no paralogues among the mammals, birds, reptiles and amphibians through searching in NCBI. However, almost all of the fish have two paralogues named *fosaa* and *fosab* except individual original fish such as the West African lungfish (*P. annectens*), Gray bichir (*P. senegalus*) and coelacanth (*L. chalumnae*). The flanking genes of *c-fos* were conserved in mammals, birds, reptiles, and amphibians, while the flanking genes of *fosaa* and *fosab* exist with deletions or other gene additions in the majority of fish (Figure 1). The multiple sequence alignments of vertebrate Fos indicated that amino acids are not completely conserved, and some relatively conserved amino acid sites altered mainly in fish (Figure 2). To investigate the evolutionary relationships of Fos among the vertebrates, we constructed the phylogenetic trees using amino sequences. The results showed that the Fos protein diverged into two groups, which the mammals, birds, reptiles and amphibians were grouped together with the fosab protein of fish, and the fosaa protein of fish stands alone as a group. The Fos protein of mammals is increasingly distant from that of birds, reptiles, amphibians, and fish in kinship ordering, which could be used as identity genes in evolution. Besides, the fish species with one paralogue named fosab have a higher amino acid sequence similarity to Fos in mammals and amphibians (Figure 3).

### 3.2 Generation of *fosaa* and *fosab* knockout zebrafish

**FIGURE 4:**
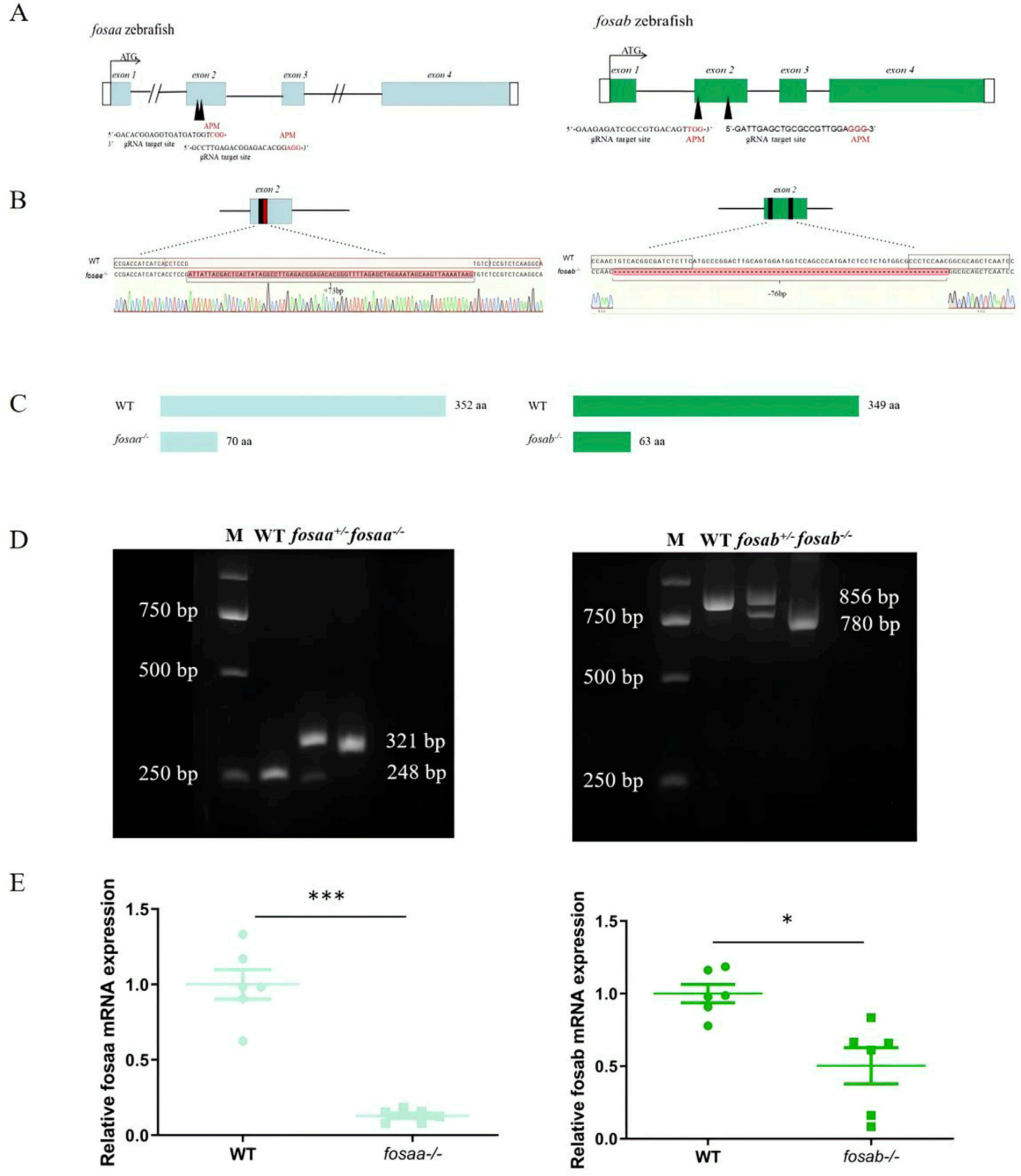
Generation of *fosaa* ^*−/−*^ and *fosab* ^*−/−*^ zebrafish using CRISPR/Cas9. **(A)**: Schematic representation of gene structure for *fosaa* and *fosab*. The open reading frame (ORF) of *fosaa* and *fosab* are respectively indicated by blue and green boxes. The black lines show untranslated introns. The write boxes show regions. The translation start site (ATG) is indicated by a broken line. Both *fosaa* gene and *fosab* gene have 4 exons and the target sites are located in exon 2 by black arrowhead. The black and red letters respectively represent the target region and protospacer adjacent motif (PAM). **(B)**: Mutation pattern of *fosaa* and *fosab* knockout. The black and red boxes represent sgRNA target binding site and PAM respectively. The red letters and hyphens represent nucleotide insertion and deletion. **(C)**: The *fosaa* and *fosab* deletions and insertions caused frame-shift early termination of translation, resulting in *fosaa* ^*−/−*^ and *fosab* ^*−/−*^ proteins with only 70 and 63 amino acids (aa) individually. **(D)**: Left and right panels show PCR genotyping of *fosaa* and *fosab* knockout zebrafish. WT, ^+/−^ and ^−/−^ indicate wild-type, heterozygous and homozygous zebrafish, respectively. **(E)**: Relative mRNA expression of *fosaa* and *fosab* in WT zebrafish, *fosaa* ^*−/−*^ and *fosab* ^*−/−*^ zebrafish. Data represent mean ± SEM (n = 6). Values marked with * is significantly different with the WT group (*P* < 0.05) and *** is significantly different with the WT group (*P* < 0.001).

We constructed the *fosaa* and *fosab* mutants using the CRISPR/Cas9 technique in zebrafish, and sgRNA is located at the second exon adjacent motif (APM) individually (Figure 4A). The *fosaa* and *fosab* homozygous mutants (*fosaa* ^−/−^ and *fosab* ^−/−^) resulted in a frameshift and premature truncation of proteins with 73 bp deletion and 76 bp insert, respectively (Figures 4B, C). F1 heterozygous were crossed to produce F2 homozygous and generated F3 stable genetic mutant lines (Figure 4D). The knockout zebrafish are viable and fertile. Further RT-qPCR analysis showed that *fosaa* ^−/−^and *fosab* ^−/−^ mRNA expression was significantly decreased compared with wild-type (WT) (Figure 4E).

### 3.3 Brain weight is reduced in adult *fosaa* ^−/−^ and *fosab* ^−/−^ zebrafish

**FIGURE 5:**
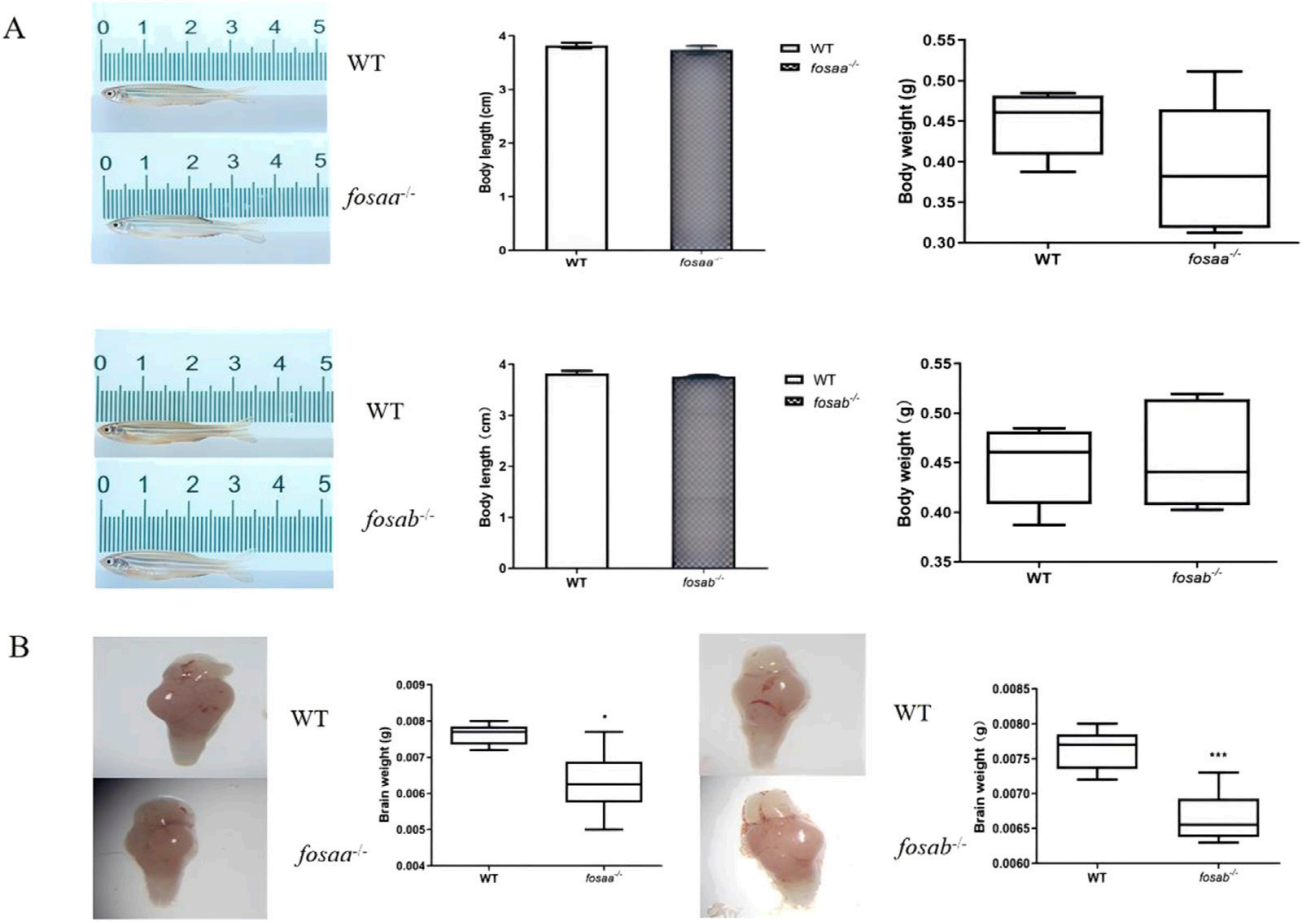
Growth performance and brain weight were observed in *fosaa* ^−/−^, *fosab* ^−/−^ and WT groups. **(A)**: Body length and body weight of *fosaa* ^−/−^, *fosab* ^−/−^ and WT groups. **(B)**: Brain weight of *fosaa* ^−/−^, *fosab* ^−/−^ and WT groups. The data in the figure were expressed as mean ± S.E.M. (n = 6), and values marked with * is significantly different with the WT group (*P* < 0.05) and *** is significantly different with the WT group (*P* < 0.001).

The growth performance was observed in adults *fosaa* ^−/−^, *fosab* ^−/−^ and WT zebrafish, and the results indicated that there was no difference with body length and weight (*P* > 0.05) (Figure 5A). The brain weight showed a marked reduction in *fosaa* ^−/−^ (*P* < 0.05) and *fosab* ^−/−^ (*P* < 0.001) groups compared with the WT group (Figure 5B).

### 3.4 T-maze test

**FIGURE 6:**
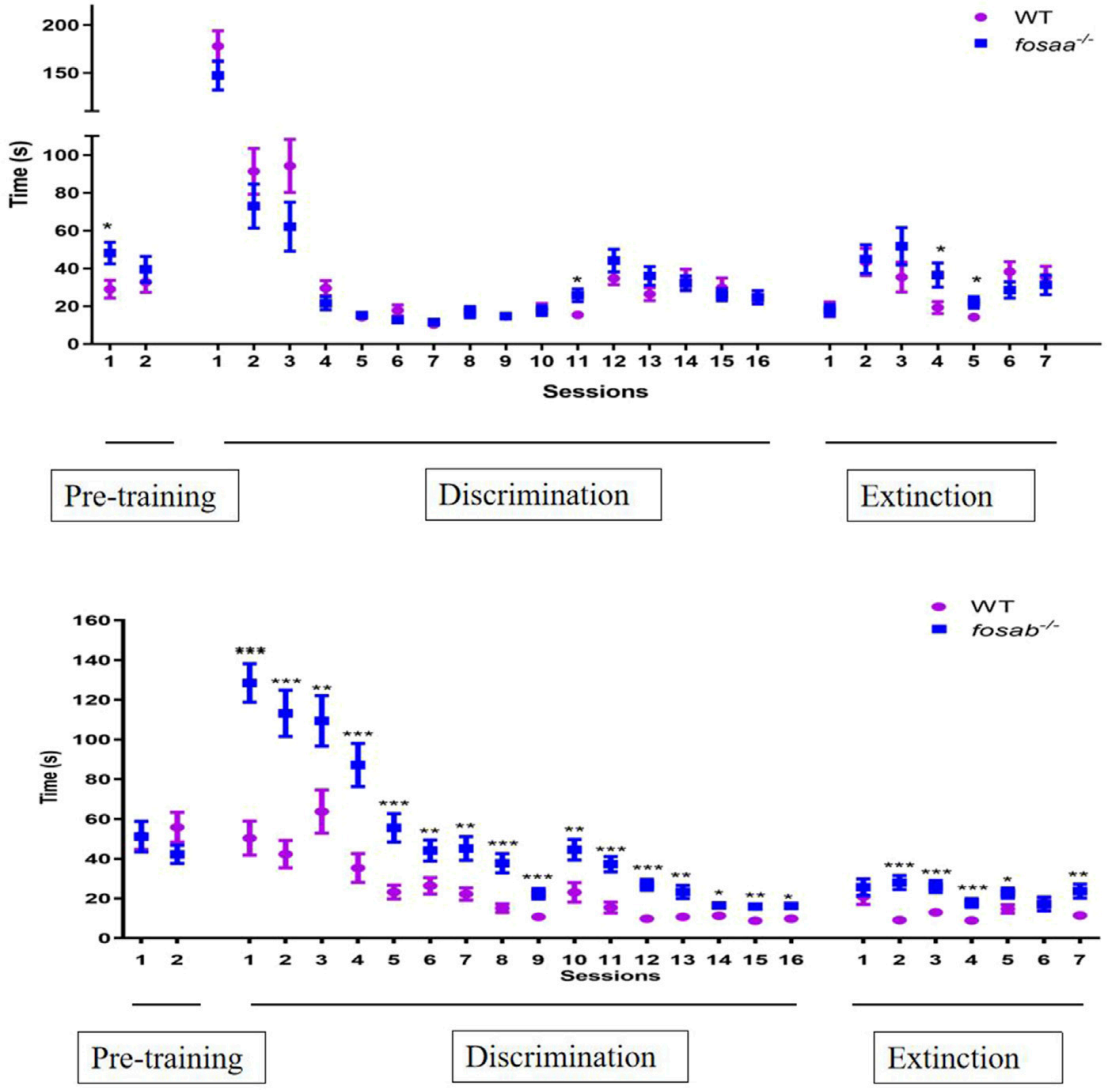
The average time required by zebrafish to complete trials in pre-training (2 days), discrimination learning training (16 days) and decision test (7 days). The data represented by mean ± SEM (n = 6). *, **, *** indicated significant differences (*P* < 0.05) (*P* < 0.01) (*P* < 0.001) compared with the WT group at the same session respectively.

The average time taken for *fosaa* ^−/−^, *fosab* ^−/−^ and corresponding WT zebrafish to accomplish the trial for each session were shown in Figure 4. There was no significant difference in the average time taken for *fosaa* ^−/−^, *fosab* ^−/−^ zebrafish compared with the corresponding WT zebrafish in the 2 pretraining sessions (*P* > 0.05) except for the first day of *fosaa* ^−/−^ (*P* < 0.05). In the discrimination sessions, the WT zebrafish had a marked decrease in the average time taken to complete the trial compared with *fosab* ^−/−^ zebrafish as the increase in the days, while did not show a notable variance with the *fosaa* ^−/−^ zebrafish although it also has a decreasing trend. The average time taken to complete the trial in *fosab* ^−/−^ zebrafish was significantly higher than the WT zebrafish (*P* < 0.05, *P* < 0.01, *P* < 0.001) (Figure 6). However, the average time taken to complete the trial had no difference between *fosaa* ^−/−^ and WT zebrafish in the discrimination sessions (*P* > 0.05) (Figure 6). In the extinction sessions, the average time taken to complete the trial in *fosab* ^−/−^ zebrafish was significantly higher than the WT zebrafish, while the *fosab* ^−/−^ zebrafish had no obvious difference compared with WT zebrafish (Figure 6).

### 3.5 The acetylcholinesterase (AChE) and acetylcholine transferase (AChT) activity in the brain of *fosaa* ^−/−^ and *fosab* ^−/−^ zebrafish

**FIGURE 7:**
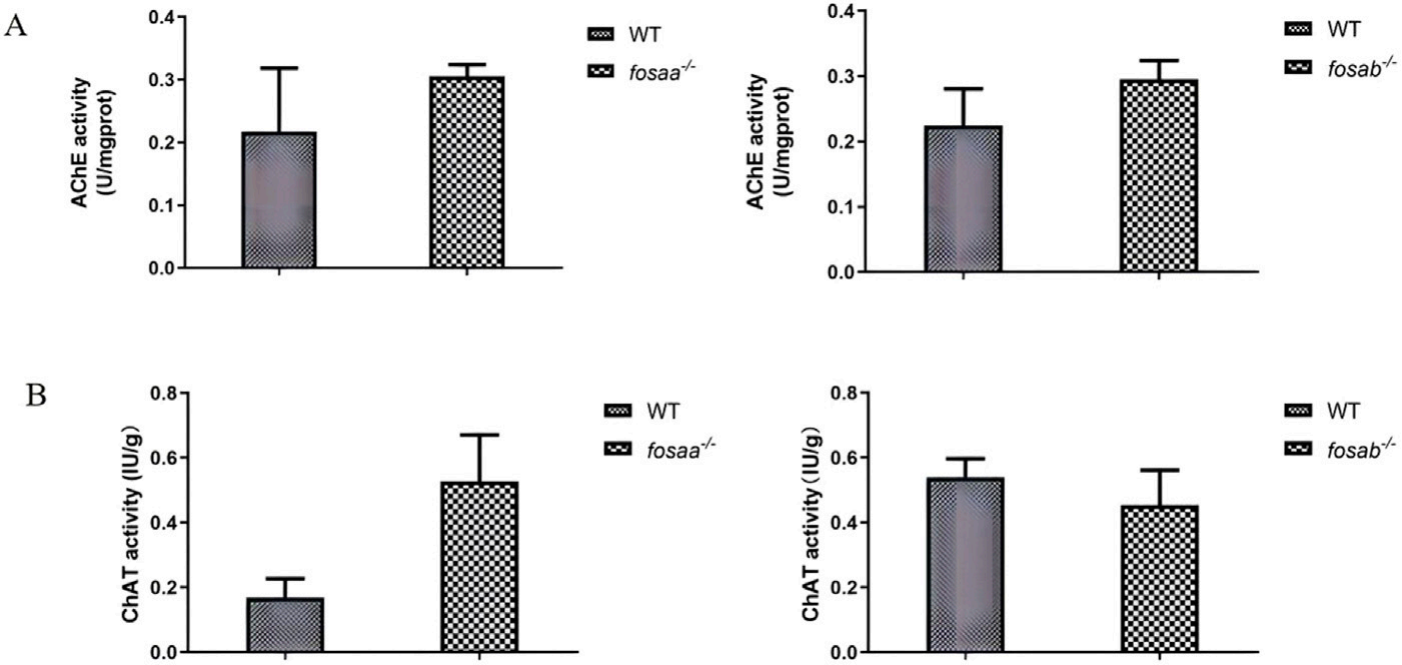
The acetylcholinesterase (AChE) acetylcholine and transferase (AChT) activity in the brain of WT, *fosaa* ^*−/−*^ and *fosab* ^*−/−*^ zebrafish. **(A)**: The acetylcholinesterase (AChE) acetylcholine in the brain of WT, *fosaa* ^*−/−*^ and *fosab* ^*−/−*^ zebrafish. **(B)**: The transferase (AChT) activity in the brain of WT, *fosaa* ^*−/−*^ and *fosab* ^*−/−*^ zebrafish. The data in the figure were expressed as mean ± S.E.M. (n = 3).

The acetylcholinesterase activity of the *fosaa* ^−/−^ and *fosab* ^−/−^ zebrafish were not significantly different from that of the WT (*P* > 0.05) (Figure 7A). By detecting the activity of acetylcholine transferase, it was found that there was no significant difference in the activity of acetylcholinesterase between the *fosaa* ^−/−^ and *fosab* ^−/−^ zebrafish compared with the WT zebrafish (*P* > 0.05) (Figure 7B).

### 3.6 The relative mRNA expression of *bdnf* and *c-jun* in the brain of *fosaa* ^−/−^ and *fosab* ^−/−^ zebrafish

**FIGURE 8:**
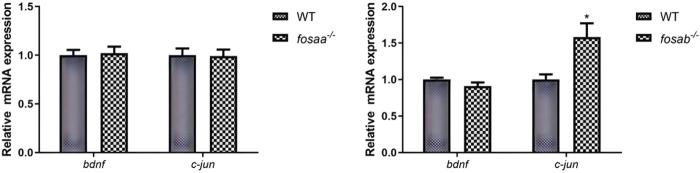
Relative mRNA expression of t of *bdnf* and *c-jun* in the brain of *fosaa* ^*−/−*^ and *fosab* ^*−/−*^ zebrafish. Data represent mean ± SEM (n = 6). Values marked with * are significantly different with the WT group (*P* < 0.05).

To evaluate how the *bdnf* and *c-jun* expression was altered in the *fosaa* and *fosab* knockout zebrafish, the relative mRNA expression of *bdnf* and *c-jun* was detected. The results indicated that the relative mRNA expression of *bdnf* and *c-jun* was not significant in *fosaa* ^−/−^ zebrafish (*P* > 0.05), while the *c-jun* in *fosab* ^*−/−*^ zebrafish was notably higher than the WT zebrafish (*P* < 0.05) (Figure 8).

## 4 Discussion

Learning and memory are prevalent in animals, from the tiny nematode worm *Caenorhabditis elegans* to humans (Johnson and Hasher, 1987; Morrison et al., 1999). Variation in learning and memory results from an adaptation to ecological conditions, which allows an individual to adapt to environmental changes (Mery, 2013). Previous studies have demonstrated that the dorsolateral telencephalon of fish could be homologous to the mammalian hippocampus, sharing functional similarities, and fish have the ability to solve complex learning tasks, while its regulatory mechanisms have not been characterized (Gómez et al., 2020; Naderi et al., 2016). The *c-fos* gene is involved in regulating learning and memory in mammals (Cai et al., 2022; Velazquez et al., 2015). However, studies on fish are limited. The *c-fos* only has one paralogue in mammals, birds, reptiles, and amphibians, while most of the fish have two paralogues, excluding individual original fish like West African lungfish (*P. annectens*), Gray bichir (*P. senegalus*), and coelacanth (*L. chalumnae*). The results indicated that there is a gene duplication of *c-fos* in the fish, which might be attributed to homologous recombination errors or large-scale duplication of chromosomal segments with whole genomes in the evolutionary process (Tian et al., 2021; Zhang et al., 2003).

To investigate whether the *c-fos* gene in fish regulates learning and memory like mammals, we utilized the zebrafish to generate *fosaa* and *fosab* zebrafish mutant lines. The mutant zebrafish did not exhibit significant difference in the length and weight compared to the WT zebrafish, which is consistent with homozygous mutant mice (Randall et al., 1992). While the brain weight of knockout zebrafish showed a significant reduction compared with WT zebrafish, and the *fosab* ^−/−^ zebrafish had a more significant decrease than the *fosaa* ^−/−^ zebrafish might be due to the *c-fos* gene assuming a functional role in the brain. Maili et al. (2023) reported that *fos* knockout in zebrafish resulted in an abnormal craniofacial phenotype characterized by a hypoplastic oral cavity and significant changes in midface dimensions. The reduced brain weight observed in *fosaa* ^−/−^ and *fosab* ^−/^ zebrafish may be attributed to these craniofacial abnormalities. The high brain weight of mice had a more rapid brain growth and behavioral maturation compared with medium and low brain, which suggests that there is a positive correlation between brain weight and brain development and learning and memory (Fuller and Herman, 1974; Paylor et al., 1994). The brain weight was reduced in the DPP6 knockout mice with slower learning and reduced memory performance in adults (Lin et al., 2018). The *c-fos* ^−/−^ mice showed a marked decrease in the brain weight resulting from a decrease in the number of cells in their organs and tissues (Velazquez et al., 2015). Thus, we figured that the *fosaa* and *fosab* knockout zebrafish could lead to impeded brain development via abnormal craniofacial and alleviating brain weight. Meanwhile, the function of learning and memory might be disrupted in the *fosaa* ^−/−^ and *fosab* ^*−/−*^ zebrafish.

The mice lacking *c-fos* in the Central Nervous System (CNS) showed impaired spatial and associative learning tasks (Fleischmann et al., 2003; Guzowski, 2002). Some behaviors that interfere with the evaluation of learning on the Morris water task were affected in the *c-fos* mutant mouse (Paylor et al., 1994). To further investigate whether the learning and memory ability of *fosaa* ^−/−^ and *fosab* ^*−/−*^ zebrafish is impaired, we performed a 24-day behavioral experiment utilizing the T-maze based on associative learning, where a visual stimulus (colour) was linked with a natural stimulus (food reward). The T-maze test can be used to perform associative learning tasks to assess the learning capabilities of zebrafish (Hieu et al., 2020; Jouandot et al., 2012; Yoo et al., 2021a). Our results found that the time both wild and knockout zebrafish took to complete a given colour choice decreased with increasing learning days in the T-maze test. It is worth noting that there is a marked decrease in the average time taken to complete the trial as the increase in the days between *fosab* ^−/−^ and WT zebrafish, while the average time taken to complete the trial had no difference between *fosaa* ^−/−^ and WT zebrafish in the discrimination and extinction sessions. The results suggested that zebrafish did have learning and memory, which may be regulated by *fosab* rather than *fosaa*. In addition to memory and learning, the *c-fos* gene is also involved in DNA damage repair, immunity, and craniofacial development (Maili et al., 2023; Yao et al., 2024; Zhang et al., 2022). *fosaa* may play an important role in other functions in fish, which deserves to be studied in depth further.

The synteny and phylogenetic analysis of *c-fos* could act as evidence for the functional differentiation of *fosaa* and *fosab*. The flanking genes of *c-fos* were conserved in mammals, birds, reptiles, amphibians, and several original fish, while the flanking genes of *fosaa* and *fosab* exist deletions or other gene additions in the majority of fish. The phylogenetic trees showed that the Fos diverge into two groups, which Fos of the mammals, birds, reptiles and amphibians were grouped together with fosab of fish, and fosaa stands alone as a group. Protein BLAST analysis showed that human c-FOS is 48% identical to Fosaa and 55% to Fosab, and the phosphorylation sites in mammalian FOS proteins are highly conserved in zebrafish Fosab but not in Fosaa (Kubra et al., 2022). Besides, (Kubra et al., 2022), observed that the pattern of fosaa and fosab expression were similar in early embryos but persisted until 4 dpf in multiple regions of the embryos via wholemount *in situ* hybridisation (WISH) analysis, which provided key insights into the tissue-specific functions of *fosaa* and *fosab*.

Fos-immunoreactive cells were identified as neurons, predominantly clustered in areas corresponding to the regions exhibiting high acetylcholinesterase (AChE) activity (Liu et al., 1991). The hyperglycemia promoted memory deficit in adult zebrafish, which results from increased AChE activity (Katiucia et al., 2014). We determined the activity of AChE and AChT in the brain of *fosaa* ^−/−^ and *fosab* ^−/−^ zebrafish, and both of them had no significant difference in *fosaa* ^−/−^ and *fosab* ^−/−^ zebrafish compared with WT zebrafish individually. Acute exposure to copper induces significant changes in the behavior of zebrafish, and the AChE activity decreased significantly in zebrafish muscle, but there were no significant changes in cerebral AChE activity (Haverroth et al., 2015). Cholinergic blocker scopolamine treatment in zebrafish has been confirmed to induce cognitive impairment, and the induced memory deficits were solely due to the drug effect on the cholinergic system (Cognato et al., 2012; Richetti et al., 2011). Gac fruit extract exhibits protective activity against scopolamine-induced memory impairment in zebrafish, while the lack of observed significant differences in AChE activity may be attributed to insufficient analysis of the adult zebrafish central nervous system (Singsai et al., 2024). Our data is in accordance with literature, indicating that the lack of differences in the enzyme activity of AChE and AChT may be attributed to the presence of several isoforms of AChE in the central nervous system and the limited number of examined samples. Therefore, whether the cholinergic system is involved in the disruption of learning and memory in the *fosaa* ^−/−^ and *fosab* ^−/−^ zebrafish requires future studies with diverse testing methods and larger sample sizes to determine.

The relative mRNA expression level of *c-jun* in *fosab* ^−/−^ zebrafish was significantly higher than in WT zebrafish, while the *fosaa* ^−/−^ zebrafish has no discernible trend. The result is consistent with our experimental results using the T-maze to assess learning and memory ability in *fosaa* ^−/−^ and *fosab* ^−/−^ zebrafish. Jun-Fos heterodimers have stronger DNA-binding activity and serve primarily as a vehicle for short-term stimulation to link synapses to long-term changes (Kuzniewska et al., 2013; Messaoudi et al., 2002). The reason for *fosab* ^−/−^ zebrafish impaired learning and memory may be via the reduction of Jun-Fos heterodimer synthesis (Cynthia and Medina, 2015). showed that BDNF is one of the upstream signals for c-Fos expression. Blocking BDNF synthesis prevents inhibitory avoidance (IA)-induced increase in c-Fos levels and affects memory persistence (Cynthia and Medina, 2015). Our results showed that the mRNA expression of *bdnf* has no significant change in *fosaa* ^−/−^ and *fosab* ^−/−^ zebrafish compared with wild zebrafish (Lucon-Xiccato et al., 2022). illustrated that zebrafish’s learning abilities were positively correlated with *bdnf* expression, and *bdnf* ^−/−^ zebrafish had a remarkable learning deficit. However, the researchers did not investigate the relationship between *bdnf* and *foaa* and *fosab* further by using the *bdnf* ^−/−^ zebrafish. In the brain of Triclocarban (TCC)-exposed zebrafish could reduce the cognitive performance of zebrafish and upregulation of *c-fos* and downregulation of *bdnf* (Lucon-Xiccato et al., 2023). Based on the available results, we hypothesized that *bdnf* might be a system that regulates learning and memory independently of *fosaa* and *fosab* in zebrafish.

In conclusion, the present study revealed that *fosab*, but not *fosaa*, may possess the function of learning and memory via synthesis heterodimers with c-jun in fish. Questions remain regarding the *fosaa* play which kind of role in fish needs to be further investigated. For the first time, we illustrated the role of *fosaa* and *fosab* in learning and memory via *c-fos* knockout in fish, which can provide new insights into environmental adaptation.

## Data Availability

The raw data supporting the conclusions of this article will be made available by the authors, without undue reservation.
