# Supplementary material for: Fosab, but not fosaa, plays important role in learning and memory in fish—insights from zebrafish gene knockout study
Source: Front Cell Dev Biol. 2025 Jan 27;13:1503066. doi: 10.3389/fcell.2025.1503066 (PMC11808014; doi:10.3389/fcell.2025.1503066)
Supplement: Supplementary file 1 [file Table1.docx]

**Supplementary table 1.** Species information.

| **Spiecies** | **Gene name** | **Accession numbers** |
| --- | --- | --- |
| *Homo sapiens* | Fos | NM_005252.4 |
| *Mus musculus* | Fos | NM_010234.3 |
| *Pan troglodytes* | Fos | NM_001098421.1 |
| *Gallus gallus* | Fos | NM_205508.1 |
| *Sus scrofa* | Fos | NM_001123113.1 |
| *Rana temporaria* | Fos | XM_040332272.1 |
| *Xenopus tropicalis* | Fos | NM_001016200.2 |
| *Taeniopygia guttata* | Fos | XM_002200534.5 |
| *Oryctolagus cuniculus* | Fos | XM_002719639.4 |
| *Phocoena sinus* | Fos | XM_032623943.1 |
| *Polypterus senegalus* | Fos | XM_039741864.1 |
| *Protopterus annectens* | Fos | XM_044073913.1 |
| *Latimeria chalumnae* | fosab | XM_005986549.3 |
| *Danio rerio* | fosaa | XM_009293306.3 |
| *Danio rerio* | fosab | NM_205569.1 |
| *Oryzias latipes* | fosaa | XM_020713725.2 |
| *Oryzias latipes* | fosab | NM_001252234.1 |
| *Fundulus heteroclitus* | fosaa | XM_036150934.1 |
| *Fundulus heteroclitus* | fosab | XM_012867394.3 |
| *Rhinichthys klamathensis goyatoka* | fosaa | XM_056241906.1 |
| *Rhinichthys klamathensis goyatoka* | fosab | XM_056269324.1 |
| *Larimichthys crocea* | fosaa | XM_027274676.1 |
| *Larimichthys crocea* | fosab | XM_010735240.3 |
| *Misgurnus anguillicaudatus* | fosaa | XM_055173241.1 |
| *Misgurnus anguillicaudatus* | fosab | XM_055186926.1 |
| *Periophthalmus magnuspinnatus* | fosaa | XM_033988814.2 |
| *Periophthalmus magnuspinnatus* | fosab | XM_033991274.2 |
| *Ictalurus punctatus* | fosaa | XM_017475492.3 |
| *Ictalurus punctatus* | fosab | XM_017461789.3 |
| *Oncorhynchus keta* | fosaa | XM_052496848.1 |
| *Oncorhynchus keta* | fosab | XM_035763162.2 |
| *Megalobrama amblycephala* | fosaa | XM_048190351.1 |
| *Megalobrama amblycephala* | fosab | XM_048207705.1 |
| *Pseudoliparis swirei* | fosaa | XM_056426269.1 |
| *Pseudoliparis swirei* | fosab | XM_056428219.1 |
| *Seriola aureovittata* | fosaa | XM_056393884.1 |
| *Seriola aureovittata* | fosab | XM_056393884.1 |
| *Lampris incognitus* | fosaa | XM_056295841.1 |
| *Lampris incognitus* | fosab | XM_056294402.1 |
| *Dunckerocampus dactyliophorus* | fosaa | XM_054796654.1 |
| *Dunckerocampus dactyliophorus* | fosab | XM_054760990.1 |
| *Clarias gariepinus* | fosaa | XM_053509957.1 |
| *Clarias gariepinus* | fosab | XM_053491064.1 |
| *Synchiropus splendidus* | fosaa | XM_053845353.1 |
| *Synchiropus splendidus* | fosab | XM_053880880.1 |
| *Pangasianodon hypophthalmus* | fosaa | XM_026934310.3 |
| *Pangasianodon hypophthalmus* | fosab | XM_026911662.3 |
| *Dicentrarchus labrax* | fosaa | XM_051395488.1 |
| *Dicentrarchus labrax* | fosab | XM_051407926.1 |
| *Lates calcarifer* | fosaa | XM_018690060.2 |
| *Lates calcarifer* | fosab | XM_018694490.2 |
| *Syngnathus scovelli* | fosaa | XM_049741462.1 |
| *Syngnathus scovelli* | fosab | XM_049757369.1 |
| *Carassius gibelio* | fosaa | XM_052619643.1 |
| *Carassius gibelio* | fosab | XM_052586670.1 |
| *Hippocampus zosterae* | fosaa | XM_052087089.1 |
| *Hippocampus zosterae* | fosab | XM_052053048.1 |
| *Ctenopharyngodon idella* | fosaa | XM_051866934.1 |
| *Ctenopharyngodon idella* | fosab | XM_051875105.1 |
| *Thunnus maccoyii* | fosaa | XM_042388583.1 |
| *Thunnus maccoyii* | fosab | XM_042390804.1 |
| *Xiphias gladius* | fosaa | XM_040137597.1 |
| *Xiphias gladius* | fosab | XM_040126171.1 |
